# Surgical intervention for small-cell lung cancer: what is the surgical role?

**DOI:** 10.1007/s11748-012-0072-9

**Published:** 2012-05-26

**Authors:** Masayoshi Inoue, Noriyoshi Sawabata, Meinoshin Okumura

**Affiliations:** Department of General Thoracic Surgery, Osaka University Graduate School of Medicine, 2-2-L5 Yamadaoka, Suita, Osaka 565-0871 Japan

**Keywords:** Small-cell lung cancer, Surgery, Review

## Abstract

Small-cell lung cancer (SCLC) is an aggressive neuroendocrine carcinoma that accounts for approximately 10–15 % of all lung cancer cases. This histological subtype is a distinct entity with biological and oncological features differing from non-small cell lung cancer (NSCLC). Treatment is mainly performed using systemic chemotherapy, although surgery in association with chemotherapy may be indicated for a minor proportion of limited-disease cases. Since the outcomes after surgical intervention in patients with very early disease are comparable to those for NSCLC, accurate clinical staging is required, particularly in terms of nodal involvement. In addition to conventional mediastinoscopy, positron emission tomography-computed tomography and endobronchial ultrasonography guided transbronchial needle aspiration have recently become available for node diagnosis. The significance of surgery for SCLC includes local disease control and treatment for cases showing mixed histology. However, only two randomized control studies have examined the efficacy of surgery in SCLC, and both yielded negative results and are out of date. We review herein several studies concerning surgery for SCLC and discuss the results from a practical standpoint. A prospective trial performed in collaboration with pulmonologists is required to address the significance of surgery, which is a limited option in the treatment of SCLC.

## Introduction

The American Cancer Society estimated that 221,130 new cases of lung cancer of all histological types and 156,940 deaths from lung cancer occurred in the United States in 2011 [1]. Small-cell lung cancer (SCLC) accounts for 10–15 % of all lung cancers and is recognized as a high-grade malignancy with rapid growth of the primary lesion and a propensity for spread to mediastinal lymph nodes or distant organs. Since SCLC is derived from neuroendocrine cells and is biologically different from non-small cell lung cancer (NSCLC), the staging system and treatment strategy have been considered as for a distinct disorder among lung cancers. Treatment for limited disease (LD)-SCLC typically comprises 4–6 courses of systemic chemotherapy with concurrent thoracic irradiation. Prophylactic cranial irradiation is also recommended in complete responders. The standard regimen for LD-SCLC is cisplatin/etoposide, as mentioned in the National Comprehensive Cancer Network (NCCN) guideline in 2012 [2].

The Japan Lung Cancer Registry Study (JLCRS) enrolled surgical and non-surgical cases in 2002, and showed a 5-year survival rate of 14.7 % for SCLC, worse than the 46.8 % for NSCLC [3]. This outcome may reflect the higher proportion of advanced disease rather than any high-grade behavior of SCLC. Indeed, the proportions of patients at c-stage III/IV have been reported as 83.9 and 43.1 % for SCLC and NSCLC, respectively, in the literature [4]. However, radical operation is still practically performed in patients with early LD without node involvement, although some of these cases are postoperatively diagnosed with SCLC. Interestingly, JLCRS found that surgically resected cases in 2004 showed a 5-year survival rate for all resected cases of 52.6 % for SCLC, comparable to those for squamous (59.1 %), large (53.3 %), and adenosquamous cell carcinomas (50.8 %) [4]. Few clinical studies to date have examined the efficacy of surgical resection for SCLC. This review is focused on the current consensus regarding surgical intervention for SCLC.

## Diagnosis

SCLC is usually diagnosed based on immunohistochemical examination for neuroendocrine markers such as chromogranin, synaptophysin, or CD56, in addition to morphological hematoxylin-eosin staining using transbronchial biopsy specimens. Since we have a greater chance of encountering small-sized lung cancers, thanks to the widespread use of Computed Tomography (CT) screening and improvements in CT resolution, the possibility of SCLC should be always considered even among clinically early lung cancers. Significant elevations in levels of tumor markers such as pro-gastrin-releasing peptide (ProGRP) or neuron-specific enolase (NSE) are also valuable indicators. The sensitivity of ProGRP is reportedly 95 %, making this marker useful in the diagnosis of SCLC [5].

## Staging

### Staging system

SCLC has been practically classified using a two-stage system initially proposed by the Veterans Administration Lung Study Group (VALG) in 2002, as either LD confined to the hemithorax or extensive disease (ED) extending beyond the ipsilateral hemithorax [6]. The tumor, node, metastasis (TNM) system, which is generally used for NSCLC staging and is beneficial in resectable cases, has been recognized as largely ineffective for SCLC, because resectable cases represent a very minor population.

Version 7 of the TNM staging system was recently proposed by the International Association for the Study of Lung Cancer (IASLC) and 12,620 SCLC cases were analyzed [7]. Among those, 349 patients (2.8%) underwent surgical resection and the 5-year survival rates were 56, 57, 38, 40, 12, and 0 % for p-stage IA, IB, IIA, IIB, IIIA, and IIIB, respectively [8]. TNM classification could thus be of use for resectable SCLC, albeit with worse survival rates than for NSCLC. When considering surgical indications in SCLC, TNM staging could be more useful than LD/ED staging, while the problem regarding similar outcomes between p-stage IA and IB, and IIA and IIB should be resolved in the future.

### Lymph node evaluation

Pre-treatment evaluation, especially for lymph node metastasis, is quite important to decide operative indications for early SCLC. Positron emission tomography (PET) is a powerful diagnostic imaging that is reportedly useful in SCLC staging [9, 10]. Although the level of evidence is currently insufficient, PET could be useful in SCLC staging [11]. The initial staging of SCLC using PET-CT has shown 93 % sensitivity and 100 % specificity, more accurate than conventional staging with CT, bone scintigraphy, or bone marrow biopsy [12]. This suggests that PET-CT could become one of the key modalities for diagnostic imaging in the selection of resectable SCLC patients.

Pathological mediastinal node diagnosis is conventionally performed using mediastinoscopy. For approachable mediastinal lymph nodes, the sensitivity, specificity, and accuracy are 66.7, 100, and 94.6 %, respectively [13]. This study also emphasized that mediastinal metastasis (p-N2 disease) was found in 4 of 8 (50%) c-N1 cases. JCOG9101 also showed similar results for lymph node metastatic findings, with 5 of 9 (56%) c-N1 cases resulting in p-N2 postoperatively [14]. Video-assisted mediastinoscopy has recently become available and the safety of the procedure has improved. Mediastinoscopy is still a valuable procedure to rule out locally advanced LD-SCLC. Another novel technique for cytopathological node evaluation is endobronchial ultrasound-guided transbronchial needle aspiration (EBUS-TBNA). A retrospective study to investigate the utility of EBUS-TBNA in patients with SCLC demonstrated highly accurate lymph node staging with 96.4 % sensitivity, 100 % specificity, and 97.2 % accuracy [15]. The 5-year survival rate among patients selected for surgical treatment using EBUS-TBNA is reportedly 77.8 %.

## Surgical intervention

### Randomized-controlled studies

The efficacy of surgical intervention for SCLC remains controversial. Only two randomized-controlled studies have focused on addressing the significance of surgery in SCLC patients [16, 17]. The first, a landmark study by the British Medical Research Council, compared surgical resection with radiation therapy and showed no superiority of surgery [16]. After that publication, surgery for SCLC decreased and chemotherapy has played a major role in management. However, the conclusions of that investigation can no longer be considered applicable to current practice, for several reasons. Patients enrolled in the study had proximal lesions diagnosed using rigid bronchoscopy and incomplete resection was performed in half of the cases. The staging could be considered inaccurate, because the diagnostic imaging available in that era was very limited, with no CT, PET, or mediastinoscopy. The other randomized-controlled study from the Lung Cancer Study Group aimed to determine the benefits of additional surgery for residual disease following response to systemic chemotherapy using cyclophosphamide, doxorubicin, and vincristine [17]. A total of 146 patients with LD-SCLC were randomized, 70 to receive surgery and 76 to a non-surgery group. Thoracic and cranial radiation was added in both groups. Complete resection was achieved in 54 of 70 (77 %) surgery patients. Median survival time was 15.4 months for the surgery group and 18.6 months for the non-surgery group, with no significant difference observed in survival. The study concluded that additional pulmonary resection had no benefit in multimodal treatment for SCLC. Although that study enrolled only LD-SCLC cases, patients without lymph node metastasis (pN0) accounted for only 29 of 70 (41 %) surgery patients, whereas such patients are considered the main candidates for surgery at present. No documentation of outcomes for pN0 patients in the study cohort was given. Furthermore, the response rate to chemotherapy was 65 %, substantially worse than that using cisplatin/etoposide, the current standard protocol for LD-SCLC (the response rate for which is 92–97 % in association with thoracic irradiation) [18]. Thus, the implications of these two randomized-controlled studies should be carefully considered in terms of current SCLC management.

### Surgery might contribute to local control

Systemic chemotherapy using cisplatin/etoposide with concurrent thoracic irradiation is a standard treatment for LD-SCLC [19]. Although early concurrent thoracic irradiation improved survival in several studies, locoregional relapse is still frequent (26–63 %) [18, 20, 21]. Thoracic irradiation could contribute to prevent local failure, as local relapse was found in 90 % of patients treated with chemotherapy alone [22]. In addition, an investigation regarding metastatic sites using autopsy samples showed residual disease with viable cancer cells at primary tumor in 92 % of non-resected patients, while viable lesion was observed in only 31 % after radical resection [23]. The Japan Clinical Oncology Lung Cancer Study Group (JCOG) carried out a phase II trial of adjuvant chemotherapy using cisplatin/etoposide for completely resected I–IIIA SCLC [14]. The local failure rate was only 10 % in that study. Surgery might thus contribute to local control along with chemo-radiotherapy, and could thus be expected to improve survival.

### Results of surgical intervention

The results of surgical intervention for SCLC have been reported from several groups (Table 1). The University of Toronto Lung Oncology Group showed the efficacy of adjuvant surgery in addition to chemotherapy, with a 5-year survival rate of 51 % in p-stage I SCLC patients, and similar outcomes to NSCLC for p-stage II–IIIA [24]. The IASLC reported 5-year survival rates of 53 and 44 % for p-stage IA and IB, respectively [8]. Analyses of 205 patients who underwent radical lobectomy from the National Cancer Institute Surveillance Epidemiology and End Results database showed a 5-year survival rate of 50.3 % in c-stage I SCLC and better outcomes than non-surgical c-stage IA from the IASLC report [25]. They therefore recommended surgery in the treatment of localized SCLC. The Bronchogenic Carcinoma Cooperative Group of the Spanish Society of Pneumology and Thoracic Surgery (GCCB-S) presented results from a multicenter study with a 5-year survival rate of 36 % for p-stage I [26]. JCOG 9101 reported 3-year survival rates of 68, 56, and 13 % in patients with c-stage I, II, and IIIA, respectively [14]. The Thoracic Surgery Study Group of Osaka University also reported surgical results, with 5-year survival rates of 49.4 and 46.7 % in c-stage IA and IB, respectively [27]. These results are similar to those from other reports so far. Based on these historical studies, we suppose that the indication for initial surgery should be limited to LD-SCLC cases without lymph node involvement at present.

**Table 1:** Results of surgical intervention for small-cell lung cancer

Group (year)	*N*	Overall survival	Chemotherapy	Radiation
BMRC [16] (1973)	71	1YS: LD 21 %	None	None
LCSG [17] (1994)	70	2YS: LD 20 %	CPA/VCR/DXR	TRT 50 Gy
			×5 course	PCI 30 Gy
TSSGO [27] (2000)	91	5YS: c-IA 49 %, IB 47 %	Various	TRT/PCI
		p-IA 56 %, IB 30 %		
JCOG [14] (2005)	62	5YS: c-IA 66 %, IB 65 %	CDDP/VP16	None
		p-IA 73 %, IB 67 %	× 4 course	
GCCB [26] (2006)	47	5YS: p-IA/IB 36 %	Various	PCI
IASLC [8] (2009)	349	5YS: p-IA 53 %, IB 44 %	Various	Unknown
SEER [25] (2010)	247	5YS: c-IA/B 50 %	Various	Various

*BMRC* British Medical Research Council, *LCSG* Lung Cancer Study Group, *TSSGO* Thoracic Surgery Study Group of Osaka University, *JCOG* Japan Clinical Oncology Group, *GCCB* Bronchogenic Carcinoma Cooperative Group of the Spanish Society of Pneumology and Thoracic Surgery, *IASLC* International Association for the Study of Lung Cancer, *SEER* Surveillance Epidemiology and End Results, *LD* limited-disease, *YS* year survival, *CPA* cyclophosphamide, *VCR* vincristine, *DXR* doxorubicin, *CDDP* cisplatin, *VP-16* etoposide, *TRT* thoracic radiation therapy, *PCI* prophylactic cranial irradiation

### Adjuvant chemotherapy and prophylactic cranial irradiation (PCI)

The 5-year survival rate in patients who received ≥4 courses of perioperative chemotherapy was 80.8 %, significantly better than that in patients treated with 1–3 courses [27]. Addition of ≥4 courses of perioperative chemotherapy was an independent prognostic factor in multivariate analysis. Interestingly, all 4 patients with pT0N0 stage 0 who showed complete response after preoperative chemotherapy also survived more than 5 years. A prospective analysis of chemotherapy using cyclophosphamide, doxorubicin, and vincristine following surgery showed a 5-year survival rate of 50 % in p-stage I and 35 % in p-stage II [28]. According to NCCN guideline 2012, adjuvant chemotherapy alone is recommended for p-N0 SCLC patients who undergo complete resection, while concurrent chemotherapy and mediastinal radiation therapy should be considered in patients with lymph node metastasis [2]. Furthermore, PCI is currently recommended after adjuvant chemotherapy in patients with good status who undergo complete resection [2]. PCI might also contribute to the control of brain metastases, which are frequently found in patients with p-stage II–III SCLC after resection [29].

### Surgical treatment for mixed histology

Another clinically significant indication of surgery for SCLC is the treatment of mixed SCLC and NSCLC. Resected primary lesions after chemotherapy reportedly contained residual NCSLC histology in 10–25 % of patients, sometimes even in cases that had shown complete response to preoperative chemotherapy [17, 30, 31]. As residual lesions after current systemic chemotherapy using cisplatin/etoposide could contain chemo-resistant SCLC or NSCLC, surgical resection following chemotherapy is justified in such cases. We occasionally encounter combined large cell neuroendocrine carcinoma (LCNEC) as a postoperative pathological diagnosis that has been preoperatively diagnosed as SCLC based on a biopsy specimen. Although LCNEC is currently classified as a subtype of large cell carcinoma, the oncological behavior is similar to SCLC and efficacy of adjuvant chemotherapy has been reported in patients who undergo complete resection [32]. Adjuvant chemotherapy should thus be considered in patients finally diagnosed with LCNEC following surgical treatment despite preoperative diagnosis of SCLC.

In summary, the significance of surgical intervention lies in achieving local control of the primary site and treatment for NSCLC-mixed histology in SCLC. Surgery is also performed in cases with undefined preoperative pathological diagnosis. Surgery clearly has a role to play in multimodal treatment of early LD-SCLC without lymph node involvement (Table 2). Further multicenter prospective clinical trials with a large sample size are expected to be started according to evidence-based guidelines, although the accrual of such very early LD-SCLC with operative indications is extremely hard.

**Table 2:** Key notes in surgery for small-cell lung cancer

Surgery for LD-SCLC
• is aimed at achieving local control
• could be a treatment for tumor with mixed histology
• should be indicated in cases without lymph node metastasis after nodal evaluation using diagnostic imaging such as PET-CT, and mediastinoscopy or EBUS-TBNA

*LD* limited disease, *SCLC* small-cell lung cancer, *PET-CT* positron emission tomography-computed tomography, *EBUS-TBNA* endobronchial ultrasound transbronchial needle aspiration
